# A pan-cancer and single-cell sequencing analysis of CD161, a promising onco-immunological biomarker in tumor microenvironment and immunotherapy

**DOI:** 10.3389/fimmu.2022.1040289

**Published:** 2022-12-22

**Authors:** He Li, Ke Zhou, Kaiyue Wang, Hui Cao, Wantao Wu, Zeyu Wang, Ziyu Dai, Shi Chen, Yun Peng, Gelei Xiao, Peng Luo, Jian Zhang, Zaoqu Liu, Quan Cheng, Hao Zhang

**Affiliations:** ^1^ The Animal Laboratory Center, Hunan Cancer Hospital and The Affiliated Cancer Hospital of Xiangya School of Medicine, Central South University, Changsha, China; ^2^ School of Medicine, Hunan Normal University, Changsha, China; ^3^ Department of Neurosurgery, Xiangya Hospital, Central South University, Changsha, China; ^4^ National Clinical Research Center for Geriatric Disorders, Xiangya Hospital, Central South University, Changsha, Hunan, China; ^5^ Brain Hospital of Hunan Province, The Second People’s Hospital of Hunan Province, Changsha, China; ^6^ The School of Clinical Medicine, Hunan University of Chinese Medicine, Changsha, China; ^7^ Department of Oncology, Xiangya Hospital, Central South University, Changsha, Hunan, China; ^8^ Department of Geriatrics, Xiangya Hospital, Central South University, Changsha, Hunan, China; ^9^ Teaching and Research Section of Clinical Nursing, Xiangya Hospital, Central South University, Changsha, Hunan, China; ^10^ Department of Oncology, Zhujiang Hospital, Southern Medical University, Guangzhou, China; ^11^ Department of Interventional Radiology, The First Affiliated Hospital of Zhengzhou, Zhengzhou, China; ^12^ Department of Neurosurgery, The Second Affiliated Hospital, Chongqing Medical University, Chongqing, China

**Keywords:** CD161, prognosis, pan-cancer, mutation, macrophages, T cells, immunotherapy

## Abstract

**Background:**

CD161 has been linked to the appearance and development of various cancers.

**Methods:**

The mutation map and the variation of CNVs and SNVs of CD161 were displayed according to cBioportal and GSCALite. We also evaluated the pathway enrichment and drug sensitivity of CD161 according to GSCALite. We performed a single-cell sequencing analysis of cancer cells and T cells in melanoma. The cell communication patterns related to CD161 were further explored. Multiplex immunofluorescence staining of tissue microarrays was used to detect the association between CD161 expression and macrophages and T cells.

**Results:**

A high CD161 level was related to neoantigens expression, pathway enrichment, and drug sensitivity. In addition, single-cell sequencing analysis showed that CD161 was mainly expressed in T cells, M1 and M2 Macrophages, neoplastic, microglial cells, neurons, and cancer cells in many tumor types. Further study on pseudotime trajectories and functional annotation of CD161 proved the critical role of CD161 in tumor progression and T cell immunity in melanoma. Multiplex immunofluorescence revealed that CD161 is closely correlated with the immune infiltration of T cells and macrophages in multiple cancers. In addition, high CD161 expression predicted a favorable immunotherapy response.

**Conclusion:**

CD161 is involved in the immune infiltration of T cells and macrophages and might be a promising target for tumor immunotherapy.

## Introduction

Cancer immunotherapy has shown critical clinical benefits in different malignancies, and more and more clinical oncologists have turned their attention to immune-oncology (1). The molecular characteristics of tumor cells and tumor-related cells as biomarkers of clinical outcomes have excellent prospects for development (2). Currently, numerous cancer patients have received immunotherapy, in which immunotherapy drugs could inhibit immune checkpoint molecules, the mediator that facilitates tumor cells’ immune escape. Programmed death-1/programmed cell death ligands (PD1/PD-Ls) have been the most important immune checkpoints (3). The American Food and Drug Administration (FDA) has already approved three new melanoma immune checkpoint drug treatments (4). These treatments may be better developed in the future.

NK receptor-P1A (NKRP1A), or killer cell lectin-like receptor subfamily B member 1 (KLRB1), is a gene encoding human CD161. The killer cell lectin-like receptors are responsible for ligand recognition and contain a C-type lectin-like domain in the extracellular region. CD161 is one of this receptor family (5). CD161 is usually expressed in monocytes, natural killer (NK) cells, NKT cells, and 25% peripheral blood T-lymphocyte (6). This C-type lectin receptor CD161 is expressed in all lineages of human T lymphocytes. The high expression level of CD161 is mainly composed of a population of mucosal-associated invariant T (MAIT) cells (7). A study showed that the expression level of CD161 mRNA in CD4+ cells in peripheral blood of tumor patients was significantly higher than that of healthy people, and the frequency of CD4+CD56− cells expressing CD161 in CD4+ T cells in peripheral blood and tumor-involved sites of cancer patients increased. It is assumed that the increase in CD4+CD161+ cells may be directly associated with the clinical status and tumor burden of patients (8). One study described that CD161 binds LLT1 with entropically and enthalpically driven thermodynamics, fast kinetics, low affinity, and small heat capacity; there are typical cell-cell recognition interactions (5). When the interaction between CD161 on NK cells and LLT1 on target cells, NK-mediated cytotoxic response will be inhibited. This interaction phenomenon has been discovered in prostate cancer, non-small cell lung cancer (NSCLC), and triple-negative breast cancer (9–11). A new study suggests that KLRB1 gene inactivation or antibody-mediated KLRB1 blockade strengthens T cell-mediated killing of glioma cells in extracorporal and their antitumor function *in vivo*. They defined the CD161-CLEC2D pathway as a latent target for immunotherapy of glioma and other human cancers (12).

This study integrated and analyzed the CD161 profiles, including expression, survival analysis, and single-cell sequencing. Therefore, a comprehensive analysis of the distribution of CD161 in human cancers helps comprehend the immune cell’s inherent role of CD161 in tumor immunization and its application prospect in malignant tumor-targeted therapy.

## Materials and methods

### Datasets collecting and preprocessing

Single-cell sequencing dataset of BLCA, GSE145137, Single-COAD, GSE81861, Single-cell sequencing dataset of HNSCC, GSE103322, Single-cell sequencing dataset of LIHC, GSE125449, Single-cell sequencing dataset of OV, GSE118828, Single-cell sequencing dataset of PRAD, GSE137829, Single-cell sequencing dataset of SKCM, GSE72056, Single-cell sequencing dataset of STAD, GSE183904, were downloaded from Gene Expression Omnibus (GEO) database (https://www.ncbi.nlm.nih.gov/geo/). Single-cell sequencing datasets of GBM, SCP50, and SCP393 were downloaded from Single Cell Portal platform (http://singlecell.broadinstitute.org). In addition, the Genome Sequence Archive (GSA) database was used to collect the Single-cell sequencing dataset of PAAD (CRA001160, https://ngdc.cncb.ac.cn/gsa/browse/CRA001160). The NCBI BioProject was used to collect the single-cell sequencing dataset of LUAD (#PRJNA591860).

### Expression analysis

Human cancer cell lines were downloaded from the Human Protein Atlas (https://www.proteinatlas.org) to explore the expression of CD161 in cancer cell lines and different cancer types. The OPENTARGET platform (https://www.targetvalidation.org/) combines genetics, chemical, and omics data to identify genes involved in disease and assist in systematic drug targeting and prioritization (13). The protein-protein interaction network of CD161 was downloaded from the STRING database (https://string-db.org/cgi/input.pl).

### Survival analysis

First, the critical value of CD161 was calculated from the R package survminer, the pan-cancer samples of 33 cancer types were divided into CD161-high and CD161-low groups. We investigated overall survival (OS) and disease-specific survival (DSS) differences between the CD161-high and CD161-low groups in 33 cancer types. Univariate cox regression analysis was used to explore the prognostic value of CD161 regarding OS and DSS in 33 cancer types.

### Single-cell sequencing analysis

Integration of Single-cell sequencing datasets GSE118389 and GSE75688 for BRCA was performed using the Anchors function from the R package Seurat. The quality of the ERCC genes and mitochondrial was controlled through the use of the R package Seurat (14). After scaling the data, dimensionality was reduced via principal component analysis (PCA). Cell clusters were identified using the FindClusters function. We identified tumor cells using the R package infercnv and copykat. The annotation of immune and tumor cells was based on specific markers. The UMAP function was used for dimensionality reduction of the visualization. The expression of CD161 was visualized using Vinplot, Dimplot and Featureplot. Analysis of cell-cell communication between the expression of CD161 and T cells was performed using the R package cellchat. To mine the relationship between CD161 expression and tumor cell transcription factors using the R package SCENIC.

### Multiplex immunofluorescence staining

Multiorgan cancer tissue microarray HOrg-C110PT-01 was purchased from the Outdo Biotech company (Shanghai, China). The tissue microarray has a total score of 110 and a total number of 69 cases. The Ethics Committee approved the sample collection used for the tissue microarray. We first used the Abs, including CD161 (Mouse, 1:100, ab197979, Abcam, UK), CD68 (Rabbit, 1:3000, GB113150, Servicebio, China), CD163 (Rabbit, 1:3000, 16646-1-AP, Proteintech, China), CD8 (Mouse, 1:3000, 66868-1-Ig, Proteintech, China) for immunofluorescence staining, and incubated with horseradish peroxidase-coupled secondary antibodies (GB23301, GB23303, China). The next step was tyramine signal amplification (TSA)(Servicebio, China), and the amplification reagents were CY3-TSA, FITC-TSA, 594-TSA and 647-TSA. After human antigen marking, the nucleus was coated with an anti-fading fixative containing 4', 6-diamino-2-phenylindole hydrochloride (DAPI). The Pannoramic Scanner (3D HISTECH, Hungary) was used to scan the stained slides and obtain multispectral images. DAPI emits blue light in fluorescence analysis, which is excited by UV at 330-380 nm and emitted at 420 nm. In fluorescence analysis, DAPI emits blue light through ultraviolet excitation wavelength of 330-380 nm and emission wavelength of 420 nm. CY3 has an excitation wavelength of 510 to 560 nm and an emission wavelength of 590 nm, emitting red light and labeling CD68. 594 has an excitation wavelength of 594 nm and an emission wavelength of 615 nm, emitting fuchsia light and labeling CD161. FITC has an excitation wavelength of 465 to 495 nm and an emission wavelength of 515 to 555 nm, emitting green light and labeling CD163. 647 has an excitation wavelength of 608 to 648 nm and an emission wavelength of 672 to 712 nm, emitting pink light and labeling CD8. The positive cells were quantified at the single-cell level in the multispectral images, and were analyzed using pan luminosity viewer (P.v 1.15.3) and the caseviewer (C.V 2.3, C.V 2.0) image analysis software.

### Statistical analysis

Student’ t-test was utilized to compare the difference of normal distribution variables between two groups, and one-way analysis of variance (ANOVA) was utilized to compare the difference of normal distribution variables between multiple groups. The Wilcoxon test was used to compare the differences of non-normally distributed variables between two groups, and the Kruskal-Wallis test was used to compare the differences of non-normally distributed variables between multiple groups. Pearson correlation analysis was utilized to calculate correlation coefficients. Heatmaps were generated using the R package pheatmap. P < 0.05 was considered statistically significant.

## Results

### Pan-cancer expression and methylation analysis of CD161

The studying structure are designed as follows, Most of our studies were based on 33 The Cancer Genome Atlas (TCGA) tumors, including the relationship between CD161 expression and tumor prognosis, the interactive body map of CD161 using the Gene Expression Profiling Interactive Analysis (GEPIA), the mutation of CD161 using cBioportal, gene set analysis of CD161 using GSCALite, neoantigens of CD161, gene set variation analysis (GSVA) of CD161, single-cell sequencing analysis, scRNA-seq in Skin Cutaneous Melanoma (SKCM), cell-cell communication and immunofluorescence of pan-cancer tissue sections. In addition, we analyzed the expression of CD161 in human cancer cell lines, clinical response to immunotherapies, and prediction for cellular transcription factors.

**Figure 1:**
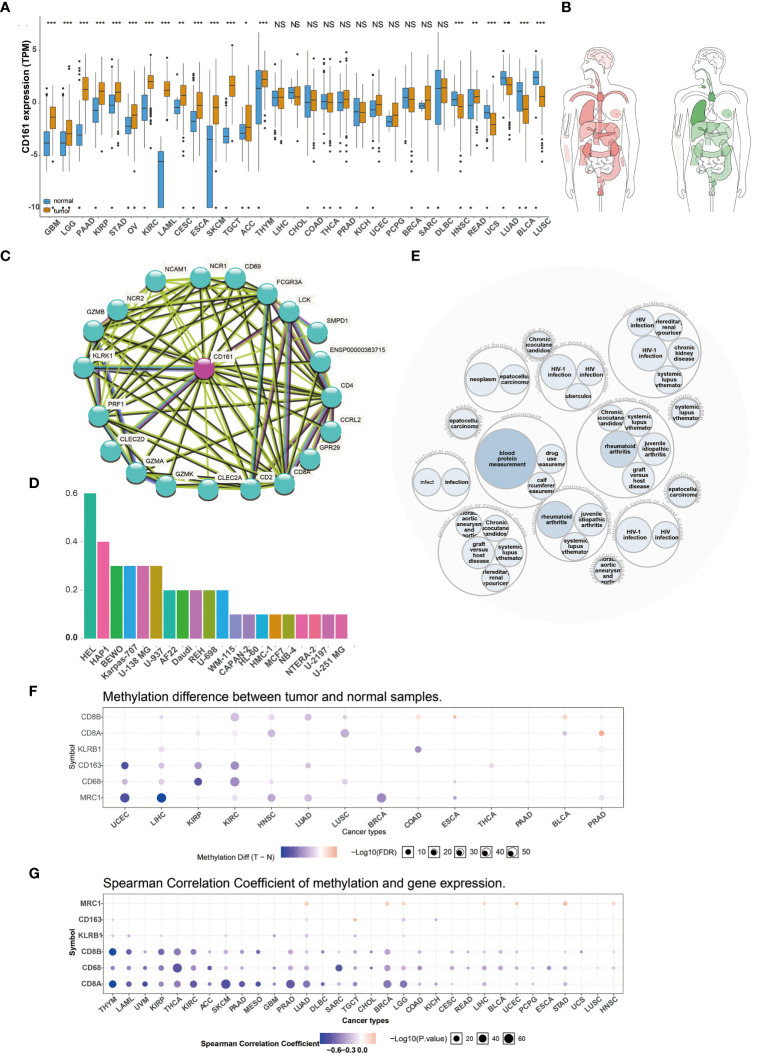
Pan-cancer Expression and Methylation Analysis of CD161. The expression of CD161 in various human cancers from TCGA dataset **(A)**. Median expression of CD161 in tissue samples in bodymap(red: tumor; green: regular) **(B)**. The protein protein interaction network related to CD161 **(C)**. The expression of CD161 in different cell lines **(D)**. A disease network interaction analysis of CD161 **(E)**. Tumor and normal tissues show differential methylation of KLRB1 and marker genes of T cells and macrophages in 14 cancer types **(F)**. Bubble map showing the correlation between the expression of KLRB1 and marker genes of T cells and macrophages and methylation across different cancer types **(G)**. ns, not statistically significant; *P < 0.05; **P < 0.01; ***P < 0.001.

We analyzed the expression levels of CD161 in multiple human cancers using TCGA (**Figure 1A**) and the interactive body map of CD161 using GEPIA (**Figure 1B**). As shown in **Figures 1A**, **B**, the Expression of CD161 was higher in various cancers, including Kidney renal papillary cell carcinoma (KIRP), Brain Lower Grade Glioma (LGG), Glioblastoma multiforme (GBM), Pancreatic adenocarcinoma (PAAD), Stomach adenocarcinoma (STAD), Ovarian serous cystadenocarcinoma (OV), Acute Myeloid Leukemia (LAML), Kidney renal clear cell carcinoma (KIRC), Esophageal carcinoma (ESCA), Cervical squamous cell carcinoma and endocervical adenocarcinoma (CESC), SKCM, Adrenocortical carcinoma (ACC), Testicular Germ Cell Tumors (TGCT), Thymoma (THYM) and Rectum adenocarcinoma (READ) than normal tissues. However, the expression of CD161 in Head and Neck squamous cell carcinoma (HNSC), Lung adenocarcinoma (LUAD), Uterine Carcinosarcoma (UCS), Lung squamous cell carcinoma (LUSC), Bladder Urothelial Carcinoma (BLCA) was lower than normal tissues. The protein protein interaction network related to CD161 based on STRING (http://string-db.org/cgi/input.pl) is shown in (**Figure 1C**). We downloaded human cancer cell lines from the human protein atlas (HPA). CD161 was highly expressed in human erythroleukemia cell line HEL, chronic myeloid leukemia (CML)-derived cell line HAP1, human placental choriocarcinoma cell line BeWo, human plasmacytoma cell line Karpas-707, glioblastoma cell line U-138 MG, human hematopoietic cell line U-937 and other cell lines (**Figure 1D**). A disease network interaction analysis exhibited that KLRB1 has multiple gene functional partners associated with immune system disease, measurement, musculoskeletal or connective tissue disease, genetic, familial or congenital disease, urinary system disease, infectious disease or post-infectious disorder (**Figure 1E**).

Many studies indicated that abnormal DNA methylation is closely related to human cancers (15). Therefore, we used the GSCALite platform to evaluate the methylation characteristics of KLRB1 and marker genes of T cells and macrophages in TCGA cancers (**Figures 1F, G**). First, we searched for DNA methylation differences between normal and tumor tissues in 14 cancer types. This result indicated that the methylation of KLRB1 was a most pronounced decrease in Liver hepatocellular carcinoma (LIHC), Colon adenocarcinoma (COAD), and Prostate adenocarcinoma (PRAD) (**Figure 1F**). Afterward, we estimated the relationship between the expression of KLRB1 and DNA methylation in 33 cancers; the findings indicated that the expression of KLRB1 and marker genes of T cells and macrophages were principally negatively correlated with DNA methylation, and only minority positive correlations (**Figure 1G**).

### Correlation between CD161 expression and prognosis of TCGA tumor patients

**Figure 2:**
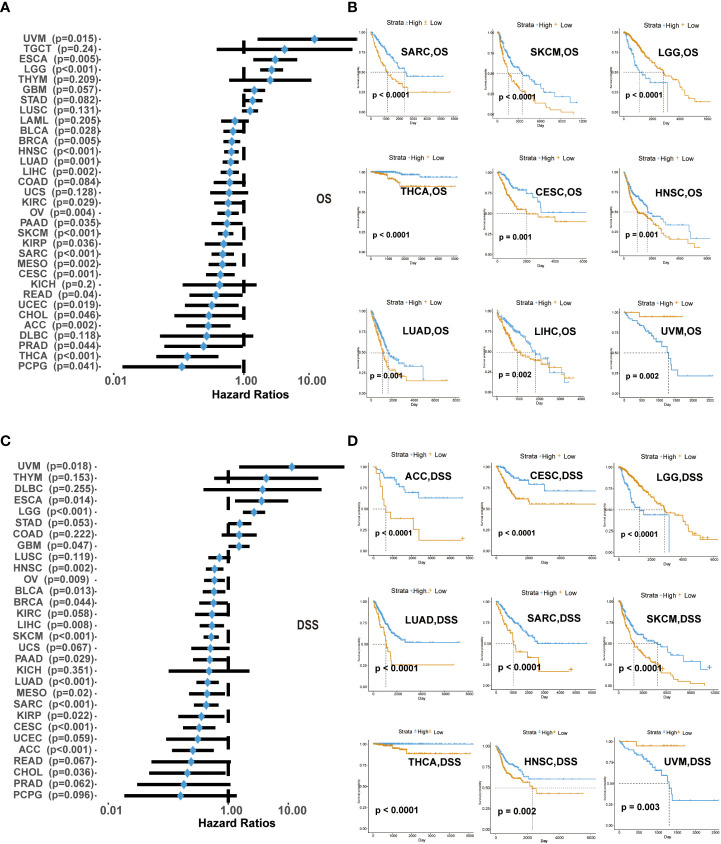
Correlation of CD161 expression with OS in 33 types of cancer **(A)**. Kaplan-Meier analysis of OS in patients with high and low expression of CD161 in SARC, SKCM, LGG, THCA, CESC, HNSC, LUAD, LIHC, and UVM **(B)**. CD161 expression with DSS in TCGA cancers **(C)**. Kaplan-Meier analysis of DSS in patients with high and low expression of CD161 in ACC, CESC, LGG, LUAD, SARC, SKCM, THCA, HNSC, and UVM **(D)**.

We explored the survival differences between CD161-high and CD161-low groups in terms of OS and DSS across 33 cancer types in TCGA. Univariate cox regression analysis was used to explore the prognostic value of CD161 regarding OS and DSS in 33 cancer types. High expression of CD161 in patients with ESCA, LGG and UVM was significantly associated with shorter OS. On the contrary, patients with low levels of CD161 expression associated with shorter OS in ACC, Breast invasive carcinoma (BRCA), BLCA, CESC, Cholangiocarcinoma (CHOL), KIRC, HNSC, KIRP, LUAD, LIHC, Mesothelioma (MESO), PAAD, OV, READ, Pheochromocytoma and Paraganglioma (PCPG), PRAD, Sarcoma (SARC), Uterine Corpus Endometrial Carcinoma (UCEC), SKCM, Thyroid carcinoma (THCA, **Figures 2A, B**, **S1**).

High expression of CD161 in patients with ESCA, LGG and UVM was significantly associated with shorter DSS, which was similar to the results of OS analysis. In contrast, low CD161 expression was associated with poor DSS in BLCA, ACC, BRCA, CHOL, CESC, GBM, KIRP, HNSC, LIHC, MESO, LUAD, OV, PRAD, PAAD, SARC, READ, SKCM, UCEC, THCA (**Figures 2C, D**, **S2**). These results indicated that CD161 expression levels were significantly associated with the prognosis of patients with multiple TCGA cancer types. High CD161 expression forecasts poor clinical outcomes in various cancer types.

### The Landscape of CD161 mutation profile in different tissues

**Figure 3:**
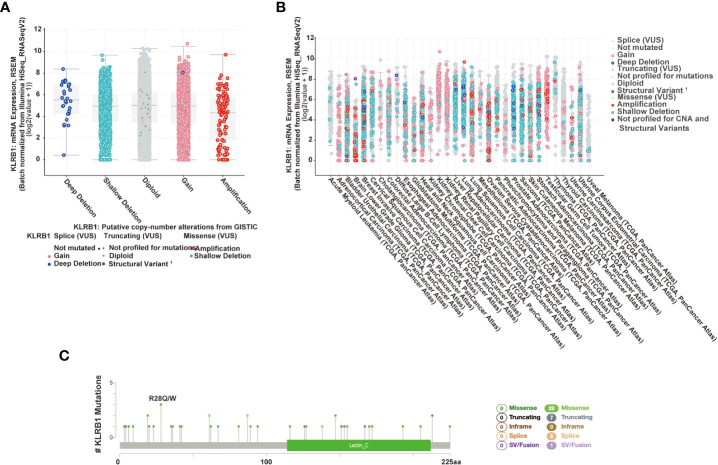
CD161 mutation landscape. The putative copy-number alterations from GISTIC of CD161 in many TCGA cancers by the cBioPortal database **(A)**. The study of the origin of CD161 in many TCGA cancers by the cBioPortal database **(B)**. Diagram of CD161 mutations across cancer types in the protein domain **(C)**.

The cBioPortal platform enables the analysis of different gene types in the TCGA database, and we used this platform to analyze the mutation frequency of CD161. We used cBioPortal to detect the mutation frequency of CD161 in the TCGA database (**Figure 3**). In addition, cBioPortal provides a different visual analysis of a single gene (**Figures 3A, B**). **Figure 3A** shows the relationship between KLRB1 mRNA expression and its putative copynumber alterations (CNAs), and **Figure 3B** shows the relationship between KLRB1 mRNA expression and its origin. A total of 42 mutation siteswere located between amino acids 0 and 225 (including 29 missenses, 7 truncating, 5 splices, and 1 fusion mutation). R28Q was the most frequent mutation site; it shows 3 of the 42 mutations in 3 patients/ samples (**Figure 3C**).

### CD161 CNVs and SNVs in different cancer types

**Figure 4:**
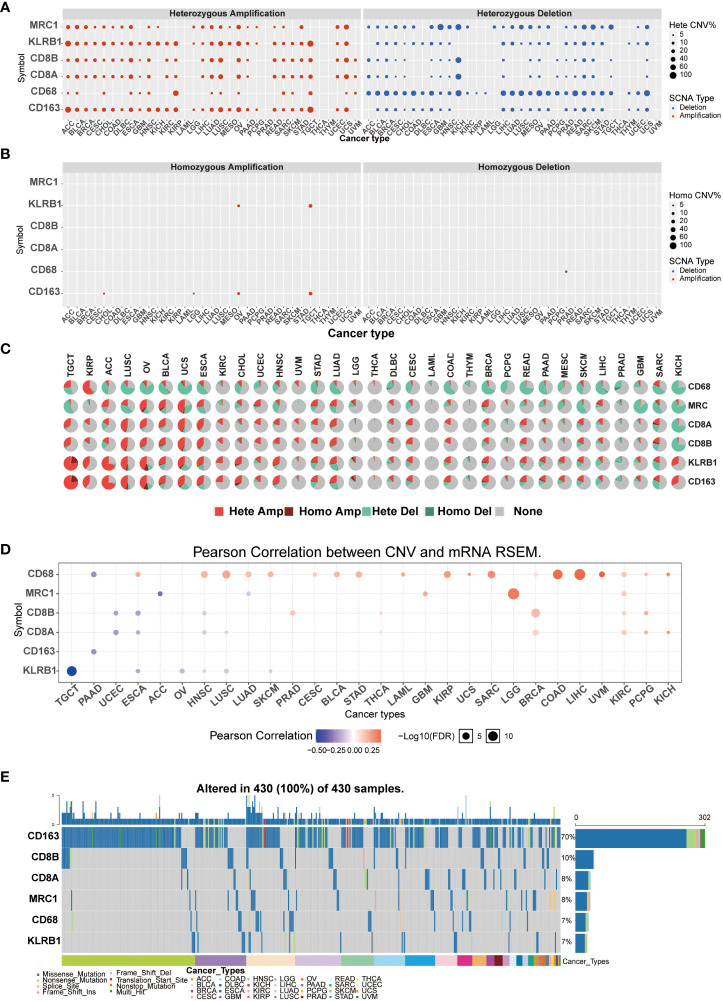
CNVs and SNVs were performed using the GSCALite platform. Heterozygous CNV of CD161 and T cells and macrophages **(A)**. homozygous CNV of CD161 and T cells and macrophages **(B)**. Pan-cancer analysis of heterozygous/homozygous CNVsof CD161 and marker genes of T cells and macrophages **(C)**. Hete Amp: heterozygous amplification; Hete Del: heterozygous deletion; Homo Amp: homozygous amplification; Homo Del: homozygous deletion; None: no CNV. The correlation between CNVs and mRNA expression levels of CD161 and marker genes of T cells and macrophages was analyzed in a pan-cancer context **(D)**. SNV frequency of CD161 and marker genes of T cells and macrophages. The gray vertical bars in the graph represent patients **(E)**. The top and side column diagrams indicate the number of variants per sample or in each gene.

Copy number variation (CNV) and single-nucleotide variant (SNV) were performed using the GSCALite platform. We predicted different types of CNA of the target genes, most CNVs are heterozygous amplification or deletion (**Figures 4A–C**). **Figure 4C** showed each cancer’s heterozygous or homozygous CNVs of CD161 and T cell and macrophage marker genes. TGCT, ACC, LUSC, OV, BLCA, USC, ESCA, LUAD, SKSM, SARC, and KICH were estimated to exhibit heterozygous amplification or deletion type CNA. In the correlation between mRNA expression and CNV, TCGA, OV, ESCA, HNSC, LUSC, and SKCM were negatively related to the expression of KLRB1 (**Figure 4D**). At least one mutation was detected in 430 samples across all analyzed tumor types. The SNV frequency of CD163 was the highest among the tumors analyzed (70%), followed by CD8B (10%). For KLRB1, SNV frequency was 7% in various cancers. In addition, the number of mutations in KLRB1-related SKCM was the largest, followed by LUSC, UCEC, and LUAD. In pan-cancer analysis, the most common DNA alterations of CD161 and the marker genes of T cells and macrophages were missense mutations (**Figure 4E**).

### The relationship between CD161 and the number of neoantigens in human cancers

Neoantigen loading may form biomarkers in cancer immunotherapy and provide the impetus for developing novel treatments that selectively enhance the reactivity of T cells to such antigens (16). Consequently, we explored the relationship between CD161 and the number of neoantigens in human TCGA cancers. Our data showed that the number of neoantigens based on SangerBox (http://sangerbox.com) was significantly related to the increase of CD161 in GBM, LUSC, COAD, and CESC (P < 0.05) (**Figure S3**).

### Drug sensitivity analysis and pathway enrichment of CD161

**Figure 5:**
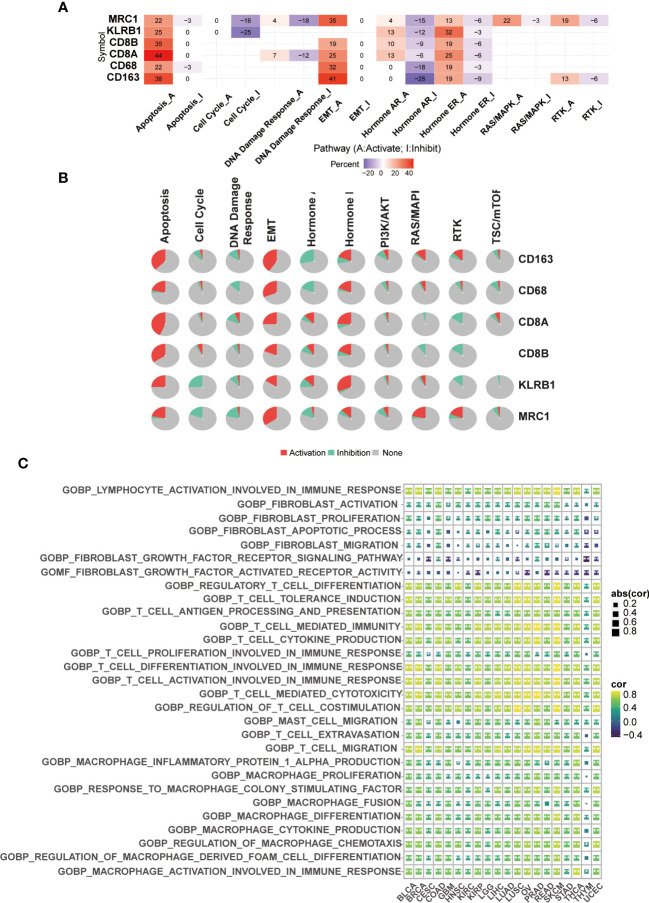
CD161 network. Venn diagram of CD161-binding genes **(A)**. Pathway enrichment of CD161 **(B)**. The GSVA algorithm was used to identify functional enrichment pathways of CD161 **(C)**. *P < 0.05; **P <0.01.

We used GSCALite for drug sensitivity analysis and pathway enrichment of CD161 and marker genes of T cells and macrophages in 33 tumors. These gene pathways are mainly related to the epithelial-mesenchymal transition (EMT), the activation of apoptosis and the inhibition of hormones AR and ER (**Figures 5A, B**). Pathway enrichment analysis based on Gene Set Variation Analysis (GSVA) algorithm showed that CD161 was active in various immune-related pathways of BRCA, BLCA, CESC, GBM, COAD, KIRC, HNSC, LIHC, KIRP, LGG, LUSC, LUAD, OV, READ, PRAD, STAD, SKCM, THYM, THCA, and UCEC (**Figure 5C**). In addition, drug sensitivity analysis showed that low-expressed CD161 exhibited resistance to 4 and 3 drugs in CTRP and GDSC, respectively (**Figure S4**).

### Single cell sequencing to reveal the expression of CD161 in tumor and immune cells

**Figure 6:**
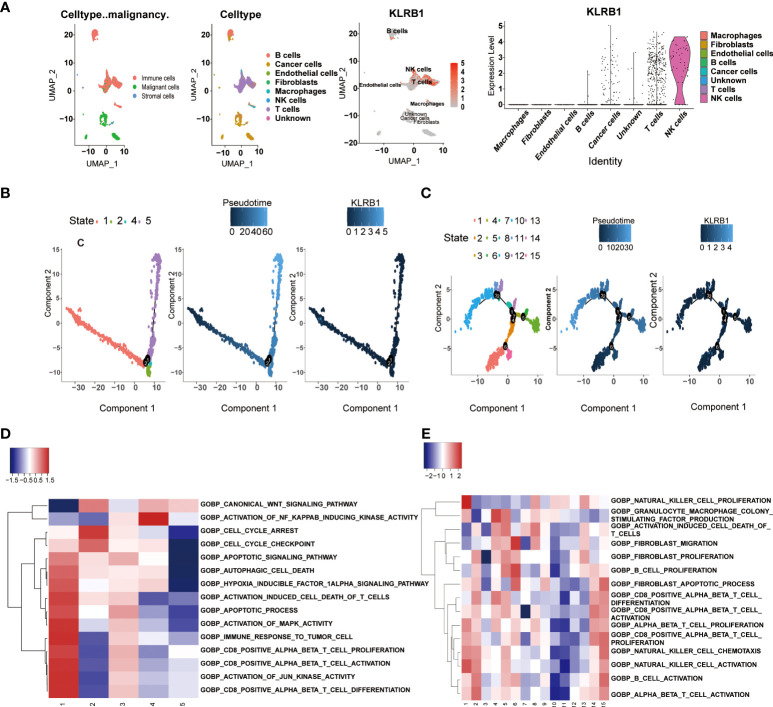
scRNA-seq results for CD161 in SKCM. The expression levels of CD161 in tumor and immune cells are based on the R package copykat in SKCM **(A)**. The single-cell trajectory of cancer cells contains three main branches. Cells in images are colored based on state (left), pseudotime (middle), and KLRB1 (right) **(B)**. The single-cell trajectory of T cells contains seven main branches. Cells in images are colored based on state (left), pseudotime (middle), and KLRB1 (right) **(C)**. GSEA of cancer cells for KLRB1 in each state **(D)**. GSEA of macrophages for KLRB1 in each state**(E)**. The red color indicates a positive correlation, while the blue color indicates a negative correlation.

Based on the R-package copykat, we studied the expression level of CD161 in tumor and immune cells in several tumor types. In LIHC, CD161 was mainly expressed in various cell types, including T cells, Thymic Epithelial Cell (TEC), B cells, Tumor-Associated Macrophage (TAM), fibroblasts, hematopoietic progenitor cell (HPC-like) and cancer cells (**Figure S5A**). Various cell types also express CD161 in LUAD and PAAD, including B cells, T cells, cancer cells, fibroblasts, macrophages, and endothelial cells (**Figures S5B, D**). Additionally, CD161 was highly expressed in NK cells in LUAD. In GBM, CD161 was highly expressed in T cells, M1 and M2 Macrophages, neoplastic, microglial cells, neurons, Astrocyte, Oligodendrocyte, Oligodendrocyte Progenitor cells, and Neural Stem cells (**Figure S5C**). In prostate cancer (PC), The expression of CD161 was the highest in T cells (**Figure S5E**). In GHOL, CD161 was mainly expressed in T cells, TEC, B cells, TAM, fibroblasts, HPC-like, and cancer cells (**Figure S5F**). CD161 was highly expressed in various cancer cells, T cells and M2 macrophages in BRCA (**Figure S6A**). In OV, CD161 was highly expressed in CD4 T cells, cancer cells, and fibroblasts (**Figure S6B**). T cells expressed high levels of CD161 in colorectal cancer (CRC, **Figure S6C**) and gastrinoma (GAS, **Figure S6D**). In head and neck squamous cell carcinoma (HNSCC), CD161 was highly expressed in Macrophages, astrocytes, T cells, M2 macrophages, Tregs, cancer cells, and fibroblasts (**Figure S6E**). Meanwhile, in SKCM, CD161 is expressed in B cells, T cells, cancer cells, and NK cells, and the expression degree is the highest in NK cells (**Figure 6A**).

### Cancer cells and T cells exhibit high CD161 expression in scRNA-Seq of SKCM

We analyzed the functional annotations and Single-cell pseudotime trajectories of cancer cells and T cells in SKCM. In cancer cells, Monocle reconstructed a trajectory, mainly including two branch points (representing “1” and “2”), and divided the cells into four states (**Figures 6B**). In T cells, Monocle reconstructed a trajectory including six branch points (denoted “1” to “6”) and divided cells into fifteen states (**Figures 6C**). The high KLRB1 expression level was observed in cancer cells in 1 and 5 states, and high KLRB1 expression was observed in T cells in 7, 8, and 9 states. Concurrently, the results showed the Gene Set Enrichment Analysis (GSEA) of KLRB1 of each “state” in cancer cells and T cells, respectively (**Figures 6D, E**). We further determined 100 genes with cancer cell branching sites; all of these genes have branching-dependent expression. We visualized the genes and related clusters expressed before and after branch points in **Figure S7A**. The top 12 genes are exhibited in **Figure S7B**. In addition, we also ascertained 100 differentially expressed genes and branch-dependent expression of branch point T cells visualized in **Figure S8A**, and the top 12 genes are exhibited in **Figure S8B**. The heat map of gene expression with pseudotime value shows that genes with similar trends converge to form different clusters. As can be seen from the figure, cancer cells and T cells are divided into three clusters (**Figures S7C, S8C**).

Moreover, the expression level of the cluster1 gene in cancer cells and T cells decreased with pseudotime. We selected six genes in cancer cells and T cells, respectively, showing their change trend with time (**Figures S7D, S8D**). The direction of dispersion between CD161 expression on tumor cells and T cells is shown in **Figures S7E, S8E**, respectively. Finally, KLRB1 was analyzed by GO enrichment analysis in cancer cells and T cells (**Figures S7F, G, S8 F, G**).

### Cell-cell communication of T cells with high and low expression of CD161

**Figure 7:**
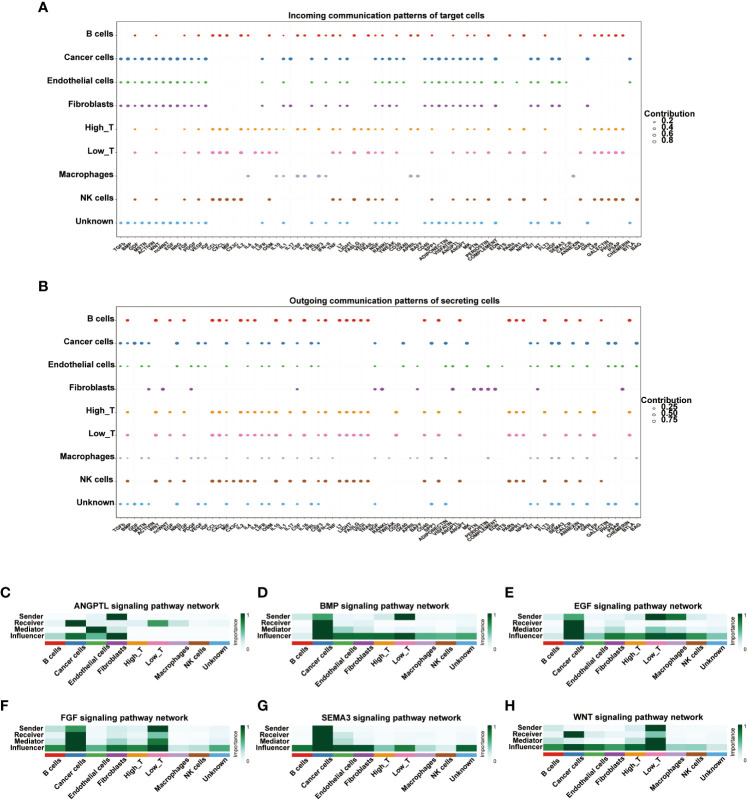
The dot plots depicted the receiver **(A)** and sender **(B)** communication patterns. The expression of CD161 is correlated with specific signal pathways, including ANGPTL **(C)**, BMP **(D)**, EGF **(E)**, FGF **(F)**, SEMAS **(G)**, and WNT **(H)**.

We used the R package “cellchat” to visualize the cell-cell communication of T cells expressing different levels of CD161. The identified roles of 9 cell types in cell-cell communication are classified into four types: receiver, sender, mediator, and influencer. The receiver and sender of these nine cell types showed three distinct cellular patterns (**Figures S9A, C**). In parallel, the specific genes associated with the receiver and sender communication patterns of the nine cell types were also divided into three distinct cell patterns (**Figures S9B, D**). The river plots depicted cancer cells, endothelial cells, fibroblasts, and unknown cells associated with the receiver’s communication patterns in pattern 1. B cells, high T cells, low T cells, and NK cells were associated with the receiver in pattern 2. Macrophages were associated with the receiver in pattern 3(**Figure S9C**). Meanwhile, B cells, high T cells, low T cells, and NK cells were associated with the communication patterns of the sender in pattern1. Cancer cells and unknown cells were associated with the sender in pattern2. Fibroblasts were associated with the sender in pattern3 (**Figure S9D**). The dot plots showed the communication patterns of the nine cell types, respectively, for the receiver and the sender (**Figures 7A, B**). We further clarified the correlations between the expression of CD161 and specific signal pathways. The results showed that T cells with high expression of CD161 interacted closely with cancer cells through signal pathways such as ANGPTL, BMP, EGF, FGF, SEMAS, and WNT (**Figures 7C–H**).

### Correlation between CD161 expression, macrophages, and T cells

**Figure 8:**
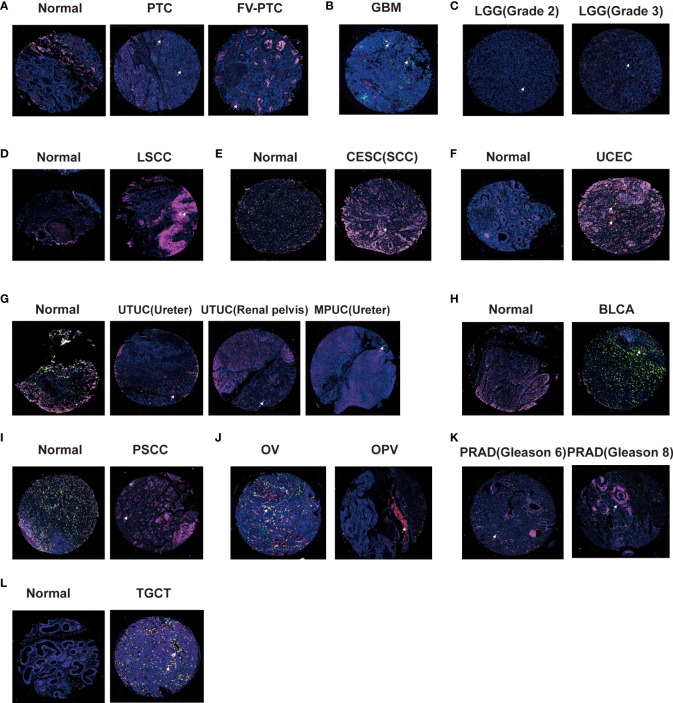
Triple immunofluorescence method was used to analyze the correlation between CD161 expression and T cells and macrophages. The expression levels of CD161 on T cells and macrophages in PTC and FV-PTC **(A)**, GBM **(B)**, LGG **(C)**, LSCC **(D)**, CESC **(E)**, UCEC **(F)**, UTUC and MPUC **(G)**, BLCA **(H)**, PSCC **(I)**, OV and OPV **(J)**, PRAD **(K)**, and TGCT **(L)**.

CD161 can be expressed in various immune cells and cancer cells in the tumor microenvironment (TME), especially T cells and macrophages. Therefore, we used tissue chips of pan-cancer samples to explore the expression profile of CD161 in macrophages and T cells. We selected CD68, CD163, and CD8 as macrophages, M2 macrophage markers, and T cell markers. Immunofluorescence showed that papillary thyroid carcinoma (PTC) and the Follicular variant of papillary thyroid carcinoma (FV-PTC) exhibit more CD161 expression than normal tissues (**Figure 8A**). Our results showed that the expression level of CD161 in WHO II gliomas was significantly lower than that in WHO III gliomas (**Figure 8C**). Meanwhile, we found that the level of CD161 shown by GBM was higher than that of LGG, and GBM was significantly expressed in M2 macrophages (**Figure 8B**). The expression of CD161 in laryngeal squamous cell carcinoma (LSCC) (**Figure 8D**), CESC (**Figure 8E**), UCEC (**Figure 8F**), and TGCT (**Figure 8L**) was higher than in normal tissues. On the contrary, the CD161 expression was decreased in tumor tissues than in normal tissues in upper tract urothelial cancer (UTUC) and micropapillary urothelial carcinoma (MPUC; **Figure 8G**), BLCA (**Figure 8H**), and penile squamous cell carcinoma (PSCC; **Figure 8I**). In addition, we compared the expression of CD161 in OV and ovarian papillary adenocarcinoma (OPV) and found that CD161 was mainly expressed in T cells (**Figure 8J**). Moreover, the expression of CD161 was up-regulated in PRAD patients with higher Gleason scores than in patients with lower Gleason scores (**Figure 8K**).

### Immunotherapy is more practical for high CD161 patients

**Figure 9:**
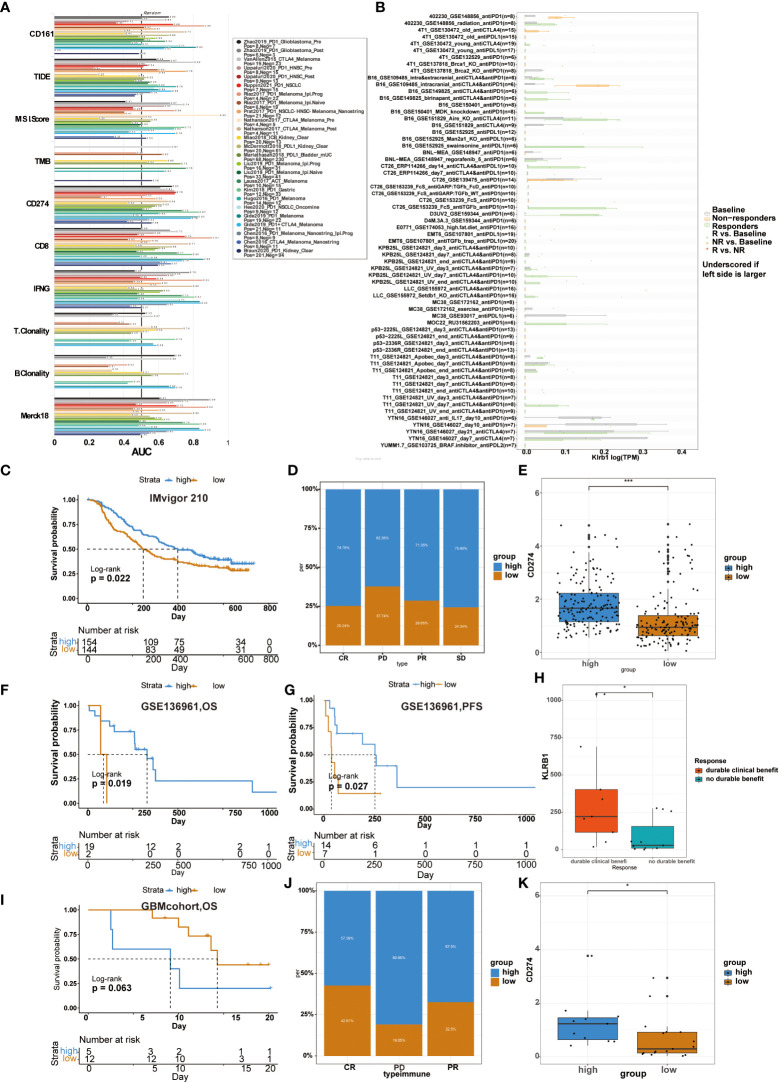
Relationship between CD161 expression and immunotherapy from two websites and four databases. Several markers area under the ROC curve (AUC) for predicting immune response **(A)**. Different ICB treatments stimulated KLRB1 gene expression in various models **(B)**. In the Imvigor 210 cohort, the correlation between patient risk score and OS **(C)**. CD161 expression correlated with the treatment intensity and response to PD-L1 immunotherapy **(D)**. Correlation between patient risk score and CD274 level **(E)**. The GSE136961 cohort shows the correlation between patient risk score and OS **(F)** and PFS **(G)**. Relationship between KLRB1 expression level and clinical benefit **(H)**. In GBM cohort, the correlation between patient risk score and OS **(I)**. In the GSE78220 cohort, CD161 expression correlated with the treatment intensity and response to PD-L1 immunotherapy **(J)**. Relationship between patient risk score and CD274 level **(K)**. *P < 0.05; ***P < 0.001.

Previous studies usually used ROC to measure the true and false-positive rates at different thresholds of TIDE prediction scores. Meanwhile, the area under the ROC curve (AUC) is used as the quality measure of prediction (17). Our results showed that the predictive value of CD161 alone had an AUC >0.7 in 4 of 25 immunotherapy cohorts (**Figure 9A**). Compared with TIDE (AUC > 0.7 in 2 immunotherapy cohorts), MSI score (AUC > 0.7 in 3 immunotherapy cohorts), TMB (AUC > 0.7 in 2 immunotherapy cohorts), T.Clonality (AUC > 0.7 in 1 immunotherapy cohorts) and B.Clonality (AUC > 0.7 in 1 immunotherapy cohorts), CD161 had a higher predictive value. Compared with CD8 (AUC > 0.7 in 7 immunotherapy cohorts), IFNG (AUC > 0.7 in 8 immunotherapy cohorts) and T cell-inflamed signature (Merck 18, AUC > 0.7 in 8 immunotherapy cohorts), CD161 had a lower predictive value. Moreover, the predictive results were similar in CD161 and CD274 immunotherapy cohorts, with four each in the cohorts with AUC > 0.7 (**Figure 9A**). In addition, we used the TISMO website to evaluate genes, pathways effectively, and immune cell infiltration in the context of Immune checkpoint inhibitor (ICB) treatment to generate the hypothesis of immunotherapeutic response (18). The picture shows how different ICB treatments stimulate KLRB1 gene expression in various models (**Figure 9B**). KLRB1 expression levels in the B16, CT26, D3UV2, KPB25L, and MOC22 models were significantly up-regulated in ICB responders but non-responders.

We analyzed the predictive value of CD161 in the anti-PD-1 cohort of urothelial carcinoma (Imvigor 210), the anti-PD-1 cohort of NSCLC(GSE136961), and GBMcohort, respectively. In Imvigor 210 cohort, high-risk scores in patients were significantly associated with longer OS (**Figure 9C**). Compared with the low CD161 group, the significant treatment intensity and response to PD-L1 immunotherapy in the high CD161 group were also confirmed (**Figure 9D**). Accordingly, the level of CD274 were higher in the high-risk score group (**Figure 9E**). In the GSE136961 cohort, patients experienced prolonged OS and PFS in the high-risk score group (**Figures 9F, G**). The expression level of KLRB1 in the patient group with durable clinical benefit was significantly higher than in the patient group without the clinical benefit (**Figure 9H**). In the GBM cohort, patients with high-risk scores had shorter OS than those with low-risk scores, but the difference was not statistically significant (**Figure 9I**). Furthermore, in the GSE78220 cohort, the proportion of high CD161 expression in the CR, PD, and PR groups was 57.39%, 80.95%, and 67.5%, respectively (**Figure 9J**). The high CD161 group showed increased expression of CD274, which resulted in a reasonable response to immunotherapy (**Figure 9K**).

### Predicting biological functions of transcription factors based on scRNA-Seq of SKCM

**Figure 10:**
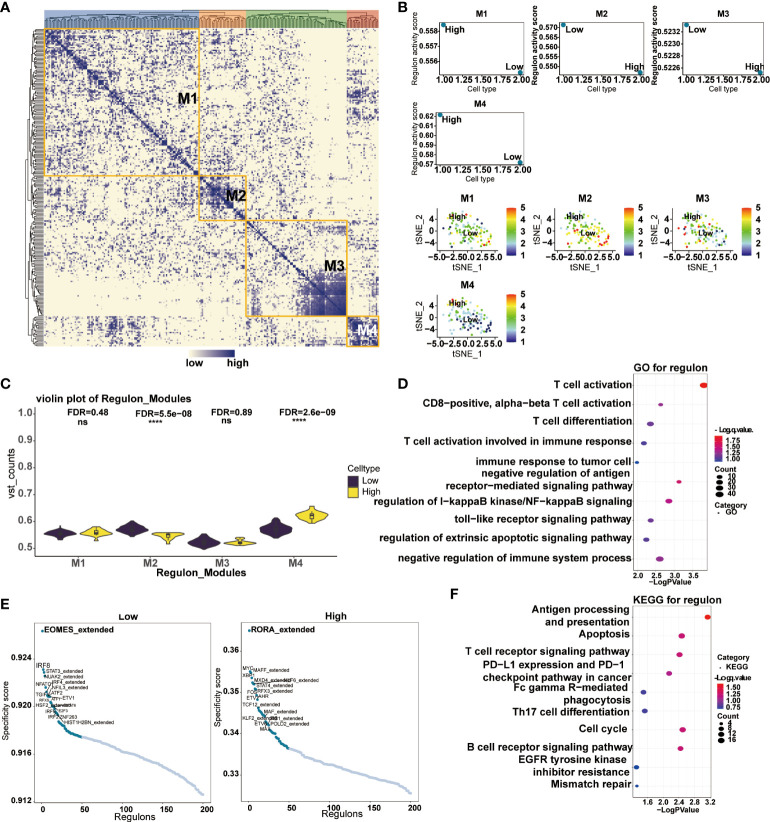
Correlation between transcription factors and CD161 expression in malignant cells. Heatmap of transcription factors in malignant tumor cells divided into four modules according to the ligation specificity index **(A)**. Regulon activity scores of transcription factors in CD161 high and CD161 low malignant neoplastic cells in four modules **(B)**. Violin plot of regulatory activity scores of transcription factors in CD161 high and CD161 low malignant cells in four modules **(C)**. GO enrichment analysis was performed to examine the possible functions of high expression regulons in malignant tumor cells **(D)**. Scatter plots based on transcription factor specificity scores in malignant cells expressed in CD161 high and CD161 low groups **(E)**. KEGG enrichment analysis was performed to examine the possible functions of high expression regulons in malignant tumor cells **(F)**. ns, not statistically significant; ****P <.0001.

Transcription factors have played critical biological functions in the tumor microenvironment. We constructed a gene regulatory system to mine the relationship between the expression of CD161 and transcription factors in tumor cells. Malignant cells were divided into four modules by the connection specificity index, in which more regulons were observed in M2 and M4 (**Figure 10A**). We then explored the interrelationship between the regulon distribution and the expression of CD161 in the four malignant cell modules (**Figures 10B, C**). The results showed that the high CD161 groups presented more regulon activities and counts in all modules, and the differences between module two and module four were significant. Subsequently, we performed GO and KEGG enrichment analysis based on the top-ranked regulons, and the results showed a high correlation between these regulons and the process of T cell activation (**Figures 10D, F**). Major specificity regulons were found in high CD161 expression tumor cells, including RORA_extended, MYC, MAFF_ extended, XBP1, MXD4_ extended, KLF8_ extended, and STAT4_ extended, while major regulons in low CD161 tumor cells including EOMES_ extended, IRF8, STAT3_extended, NUAK2_ extended, NFATC1, IRF4_ extended and NFIL3_ extended (**Figure 10E**).

## Discussion

Our results showed that the expression of CD161 is closely related to the occurrence and development of various cancers. The increasing evidence indicated that combining appropriate doses of chemotherapy with immune checkpoint inhibitors (ICIs) is more effective than monotherapy (19). Cytotoxic T-lymphocyte-associated antigen 4 (CTLA-4)/B7 and PD-1/PD-L1 are the two most crucial immune checkpoint pathways (20, 21). CD161 and its ligand LLT1 are expressed in the tumor microenvironment (11), and past studies have shown that CD161 expression on T cells can play both inhibitory and activating roles in tumorigenesis and progression (22). This study exhibited the relationships of expression levels and mutational landscape of CD161 with tumor prognosis, neoantigens, pathway enrichment, drug sensitivity, single-cell sequencing, and immunotherapy. In addition, the association of CD161 with T cells and macrophages was explored using Immunofluorescence.

First, we evaluated CD161 expression levels in 33 tumors from TCGA; it was found that there was a noticeable difference in the expression of Pan-cancer CD161 in tumor and normal tissues. TCGA data showed increased levels of CD161 in GBM, LGG, PAAD, KIRP, STAD, OV, KIRC, LAML, CESC, ESCA, SKCM, TGCT, ACC, and THYM compared to normal tissues. Several studies from us and others on CD161 showed that CD161 is highly expressed in GBM (23–25), which verified that restraining the CLEC2D-CD161 pathway may strengthen T cell-mediated immunity against GBM to a certain extent (12). Moreover, CD161 HNSC, READ, UCS, LUAD, BLCA, and LUSC was lower than normal tissues. And CD161 was expressed in 20 cell lines. DNA methylation is abnormal in all forms of cancer (26); we found that the expression levels of KLRB1 and marker genes of T cells and macrophages were principally negatively correlated with methylation and only minority positive correlations.

Studies showed that CD161 is a favorable prognostic gene whose expression primarily reflects tumor-associated leukocytes (2). Our results represented a dual role of CD161 expression in tumor prognosis, and we confirmed elevated CD161 expression with poorer prognosis in ESCA, LGG, and UVM both in OS and DSS. This result is consistent with a previous study, which indicated that the high expression of CD161 is likely to be a risk factor for patients with ESCA, LGG, and UVM (23). On the contrary, CD161 was protective in ACC, BRCA, BLCA, CHOL, CESC, HNSC, GBM, KIRP, LIHC, MESO, LUAD, PAAD, OV, READ, PRAD, SARC THCA, SKCM, and UCEC. Furthermore, we provided information about CD161 mutations, CNVs, and SNVs changes through cBioportal and GSCALite, which are discrepancies in different cancers. The mutation levels of UCS, TGCT, and OV were relatively high, and the change frequency of CD161 was more than 4%. We predicted different types of CNA of CD161 and marker genes of T cells and macrophages, most CNVs are heterozygous amplification or deletion. The SNV frequency of CD161 was 7% at the cancer level, lower than that of CD163 and CD8. The number of mutations in CD161-related SKCM was the largest, followed by LUSC, UCEC, and LUAD. The most frequent DNA alterations of CD161 and marker genes of T cells and macrophages were missense mutations. Studies showed an increase in CD8 + CD28- T cells expressing the CD161 receptor in melanoma diseases, indicating that CD8 + CD28- T cells can be activated in these patients (27, 28).

Neoantigens are produced by tumor mutations and are highly immunogenic. They are protein fragments that are not present in the normal human body. They may become a new target of tumor immunotherapy and can excitation CD8+ and CD4+ T cells to produce immune response (29). We found that the number of neoantigens was significantly related to the increase of CD161 in GBM, LUSC, COAD, and CESC. Pathway enrichment analysis showed that CD161 was active in immune-related pathways of many cancers. Furthermore, drug sensitivity analysis showed that low-expressed CD161 exhibited resistance to some drugs in CTRP and GDSC. These results emphasize that CD161 plays a complex and vital role in TME. The matrix components of TME are composed of many different cell types, such as neutrophils, cancer-related fibroblasts, myelogenous suppressor cells (30), macrophages, mast cells, regulatory T cells, natural killer cells, and platelets. These cell subsets interact with cancer cells through complex communication networks (31). Our results based on the R-package copy showed that the expression level of CD161 was extremely high in immune cells of LIHC, LUAD, GBM, PAAD, PC, and SKCM. To learn more about the role of CD161 in single-cell pseudotime trajectories and functional annotation of cancer cells and T cells in SKCM, scRNA-seq analysis was performed. In our results, we used Single-cell sequencing data to find that CD161 is highly expressed in tumor cells and T cells of SKCM. Further study on pseudotime trajectories and functional annotation of CD161 emphasizes the correlation between CD161 and T cell infiltration and progression in SKCM.

CD161 plays different roles in different cancers. Tumor-associated macrophages (TAM) and cancer-associated fibroblasts (CAF) are the most abundant noncancer cells in the tumor matrix. They have become vital participants in cancer progression, metastasis, and therapeutic resistance (32). As the core of immunosuppressive cells and cytokine network, TAM plays a crucial role in tumor immune escape (33). M1-like are activated in response to inflammatory response and antigen presentation, while M2-like can be involved in therapeutic resistance to cancer growth, metastasis, and angiogenesis (34). CD68 refers to TAMs activated by M1 and M2 macrophages, while CD163 is M2 macrophages related antigen (20, 35). Tumor-infiltrating CD8 (+) T cells can also respond in many cancers (36). In this study, we found that the expression of CD161 in PTC, FV-PTC, LSCC, CESC, UCEC, and TGCT was higher than in normal tissues. On the contrary, the expression of CD161 in UTUC, MPUC, BLCA, and PSCC was lower than in normal tissues. The multiple immunofluorescence staining results showed that CD161 was expressed in macrophages and T cells of various cancers and further verified the effects of single-cell sequencing. Therefore, we speculated that CD161 is involved in macrophage and T cell-related immune processes. It should be noted that CD161-related immune infiltration is primarily based on the interaction between CD161 located on immune cells and its ligand, presumably LLT1, located on tumor cells (37, 38).

Cancer immunotherapy recognizes and attacks cancer cells by operating the immune system, including cellular adoptive immunotherapy and immune checkpoint inhibitors therapy (39). Imvigor210 cohort and melanoma data set (GSE78220) can centrally verify the predictive value of Immune Cell Pair (ICP) score for immunotherapy response (40). We found that the predictive value of immunotherapy quality of CD161 was higher than the TIDE, MSI score, TMB, T. Clonality, and B. Clonality. Moreover, different ICB treatments stimulated KLRB1 gene expression in many models to produce a better response to checkpoint inhibitor immunotherapy. The relationship between CD161 expression and immunotherapy was obtained from four databases. Our study showed a statistically significant increase in CD274 expression in IMvigor210 and GBMcohort datasets in the high CD161 group. These results suggest that the high expression of CD161 has a reasonable response to immunotherapy, and CD161 may be involved in the immunomodulatory response of tumors by mediating immune infiltration. Although we performed relatively comprehensive single-cell sequencing and pan-cancer analysis of CD161, most of our results were based on dataset analysis, and experimental validation results were limited. Some studies have shown that the CD161-CLEC2D pathway may be a potential target of Glioma, but its effect on other cancers and the specific mechanism of action still need to be further studied.

## Conclusions

In conclusion, we provided a pan-cancer analysis of the abnormal expression, methylation, and prognosis of CD161. Although there are similar studies about CD161 in pan-cancer, our study is a more comprehensive analysis. Specifically, our study explored more the potential roles of CD161 in the tumor microenvironment of melanoma at the single-cell sequencing level and the prediction of transcription factors. CD161 is closely related to T cells and macrophages based on the multiplex immunofluorescence staining. We also analyzed the predictive value of CD161 expression in tumor immunotherapy response. Our results suggest that CD161 may be a promising target for tumor immunotherapy.

## Data availability statement

The original contributions presented in the study are included in the article/**Supplementary Material**. Further inquiries can be directed to the corresponding authors.

## Author contributions

Conception and design: HZ, KZ, KW, QC, HL. Foundation support: QC, KZ, GX, YP, HC. Acquisition and analysis of data: HZ, WW, KW, ZD, ZW. Interpretation of data: HZ, KZ, GX, JZ, PL, ZL. Drafting the manuscript and revising for submission quality: HZ, KZ. Reviewing and approving the final vision: All authors Study supervision: QC, GX, SC. All authors contributed to the article and approved the submitted version.
