# Supplementary material for: A pan-cancer and single-cell sequencing analysis of CD161, a promising onco-immunological biomarker in tumor microenvironment and immunotherapy
Source: Front Immunol. 2022 Dec 22;13:1040289. doi: 10.3389/fimmu.2022.1040289 (PMC9844218; doi:10.3389/fimmu.2022.1040289)
Supplement: Supplementary file 1 [file DataSheet_1.doc]

**Supplementary Figures 1~4:**


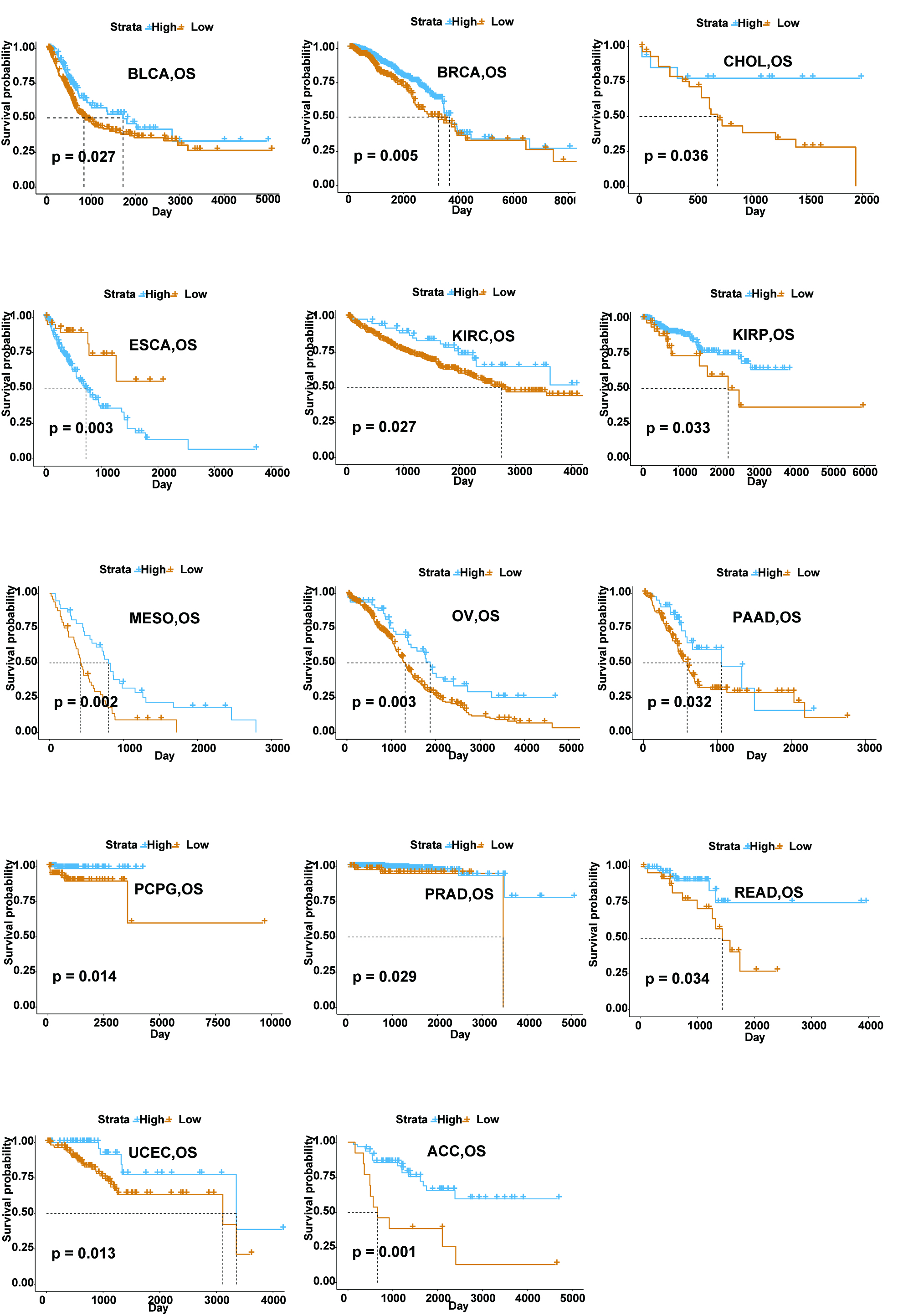


Figure S1. Kaplan–Meier analysis of OS based on high vs low expression of CD161 in ACC, BLCA, BRCA, CHOL, ESCA, KIRC, KIRP, MESO, OV, PAAD, PCPG, PRAD, READ, UCEC and (B).


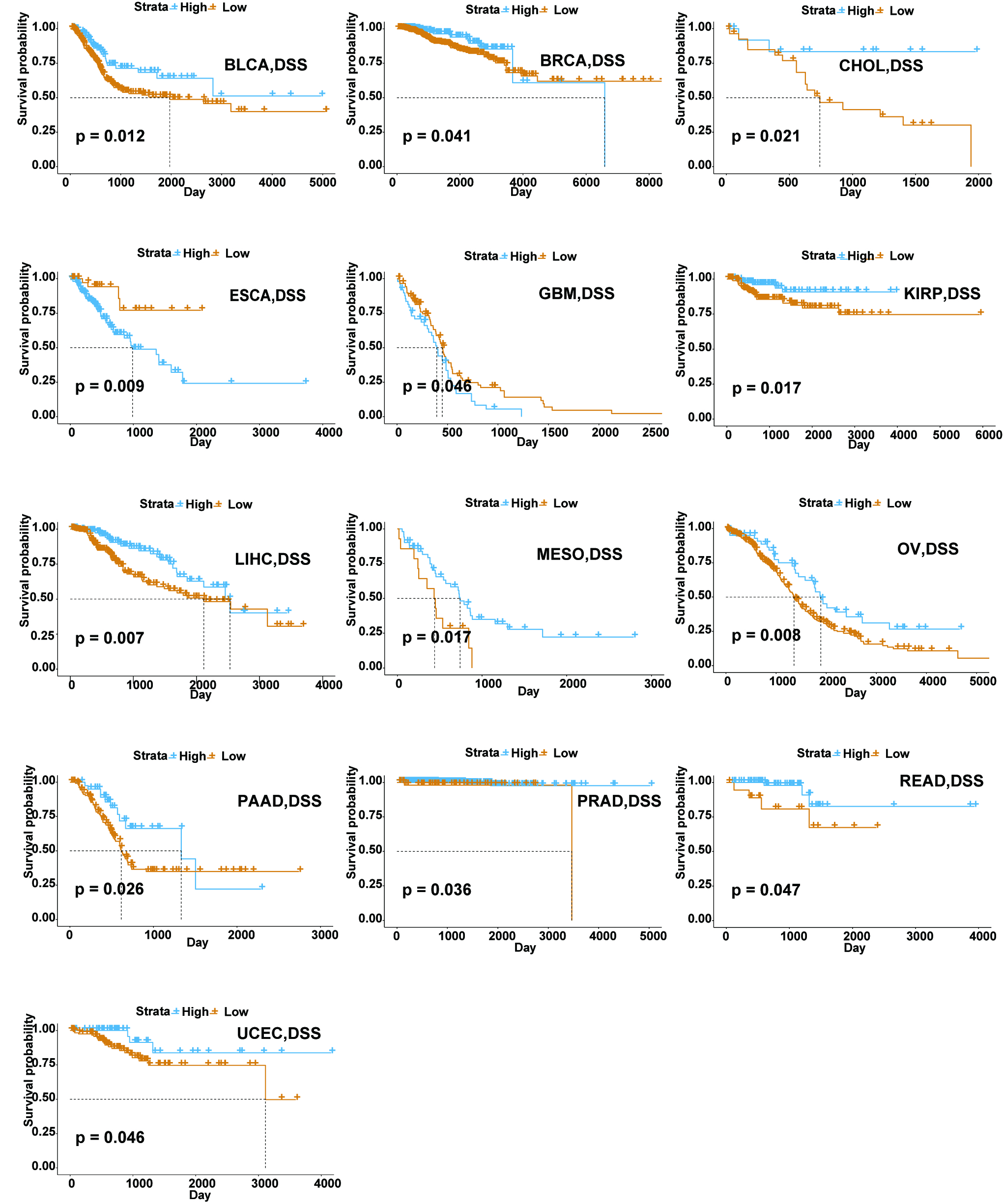


Figure S2. Kaplan–Meier analysis of DSS based on high vs low expression of CD161 in BLCA, BRCA, CHOL, ESCA, GBM, KIRP, LIHC, MESO, OV, PAAD, PRAD, READ and UCEC.


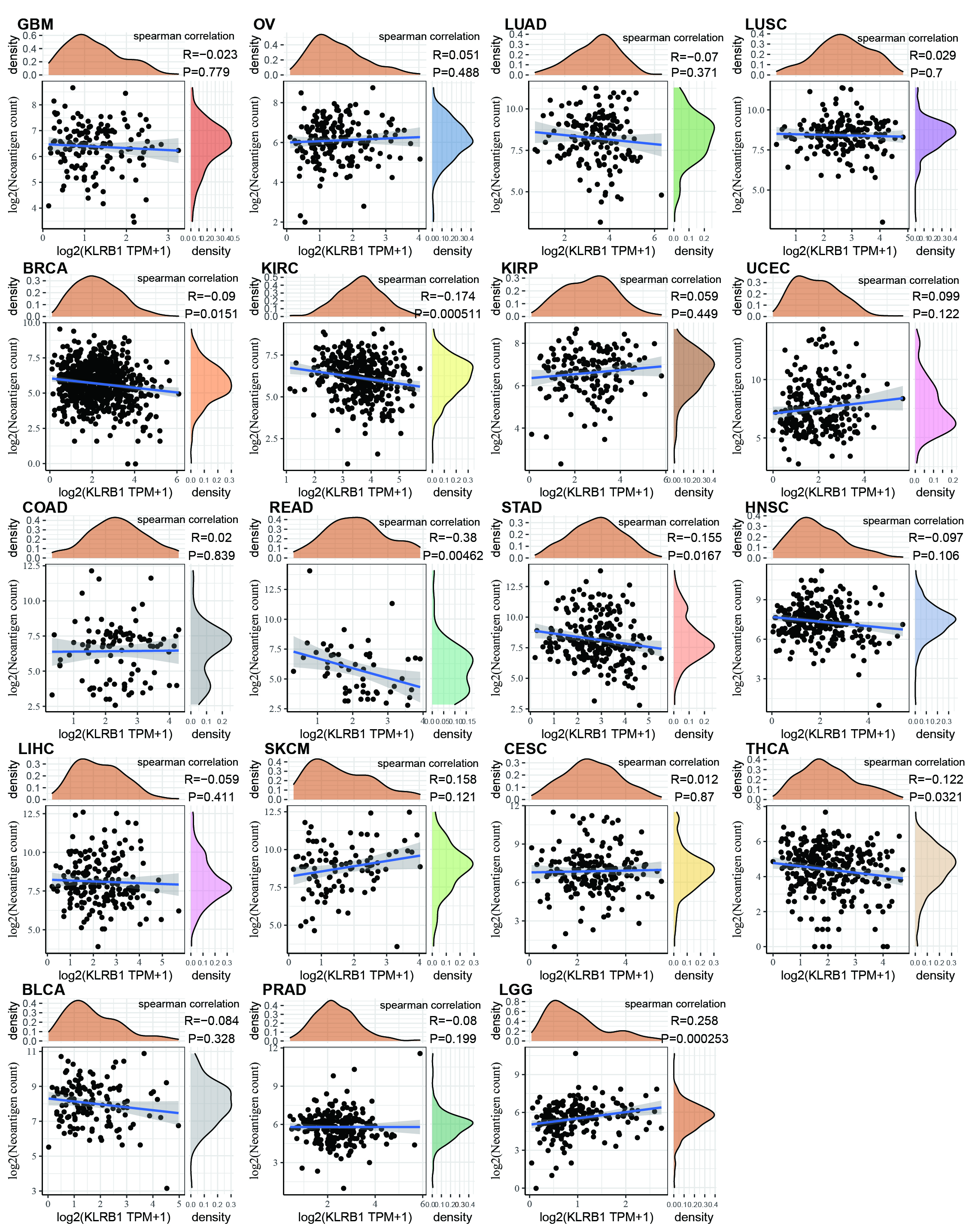


Figure S3. In pan-cancer analysis. The relationship between CD161 levels and the number of neoantigens.


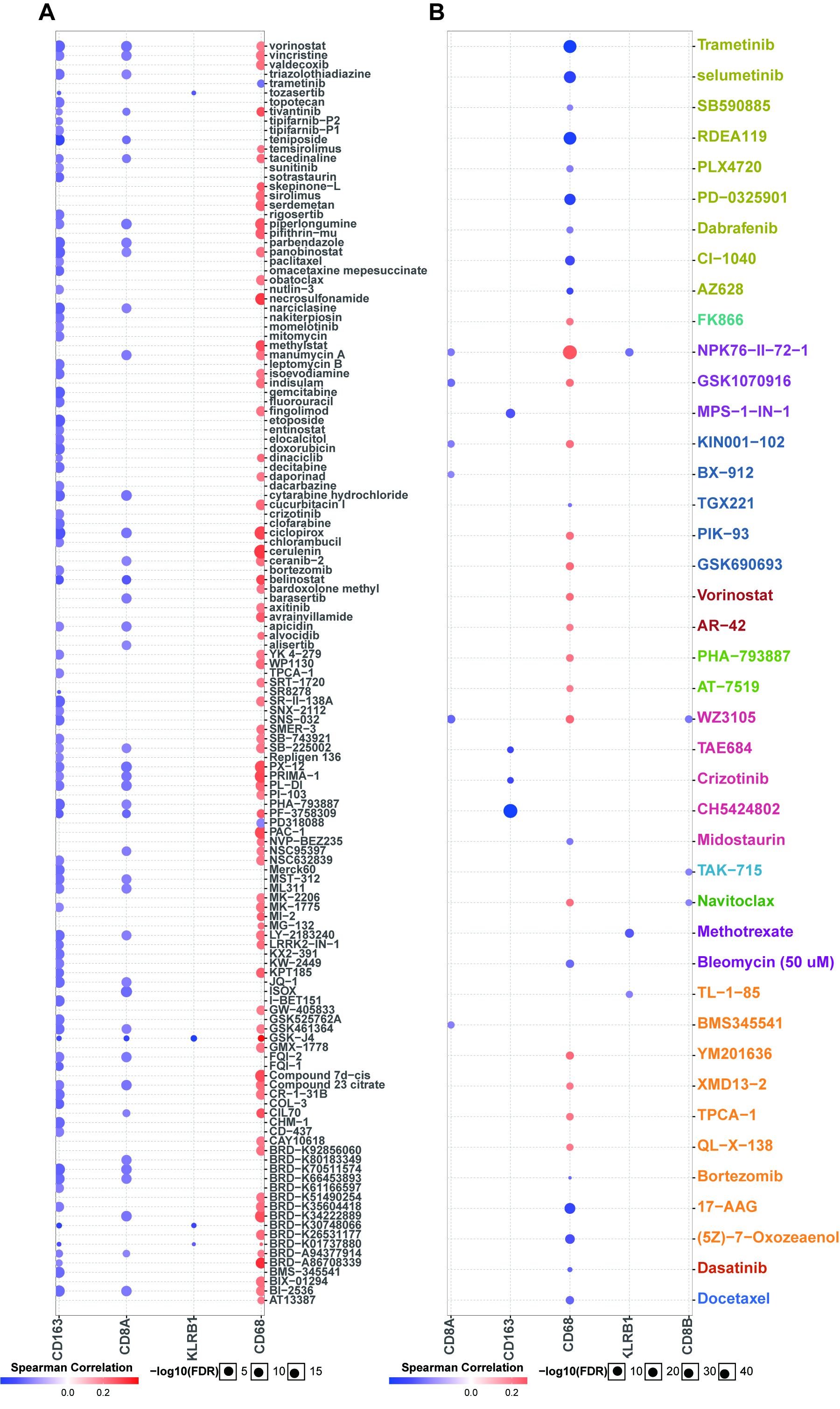


Figure S4. drug sensitivity analysis of CD161 in CTRP (A) and GDSC (B).
