# Supplementary material for: A pan-cancer and single-cell sequencing analysis of CD161, a promising onco-immunological biomarker in tumor microenvironment and immunotherapy
Source: Front Immunol. 2022 Dec 22;13:1040289. doi: 10.3389/fimmu.2022.1040289 (PMC9844218; doi:10.3389/fimmu.2022.1040289)
Supplement: Supplementary file 2 [file DataSheet_2.doc]

**Supplementary Figures 5~9:**


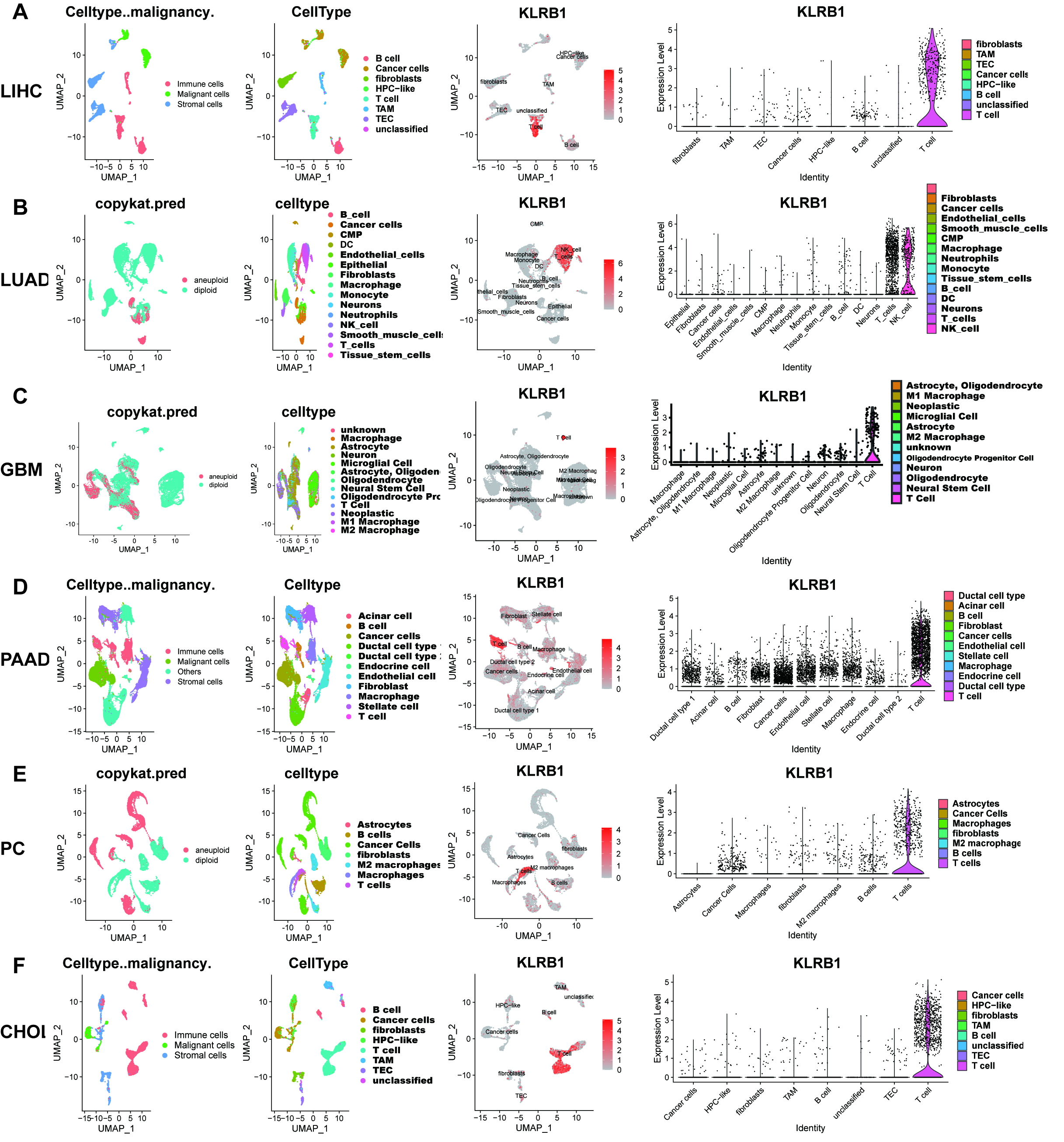


Figure S5. Single cell sequencing analysis the expression of CD161 on tumor and stromal cells. The expression levels of CD161 in tumor and stromal cells based on the R package copykat in LIHC (A), LUAD (B), GBM (C), PAAD (D), PC (E), CHOL (F).


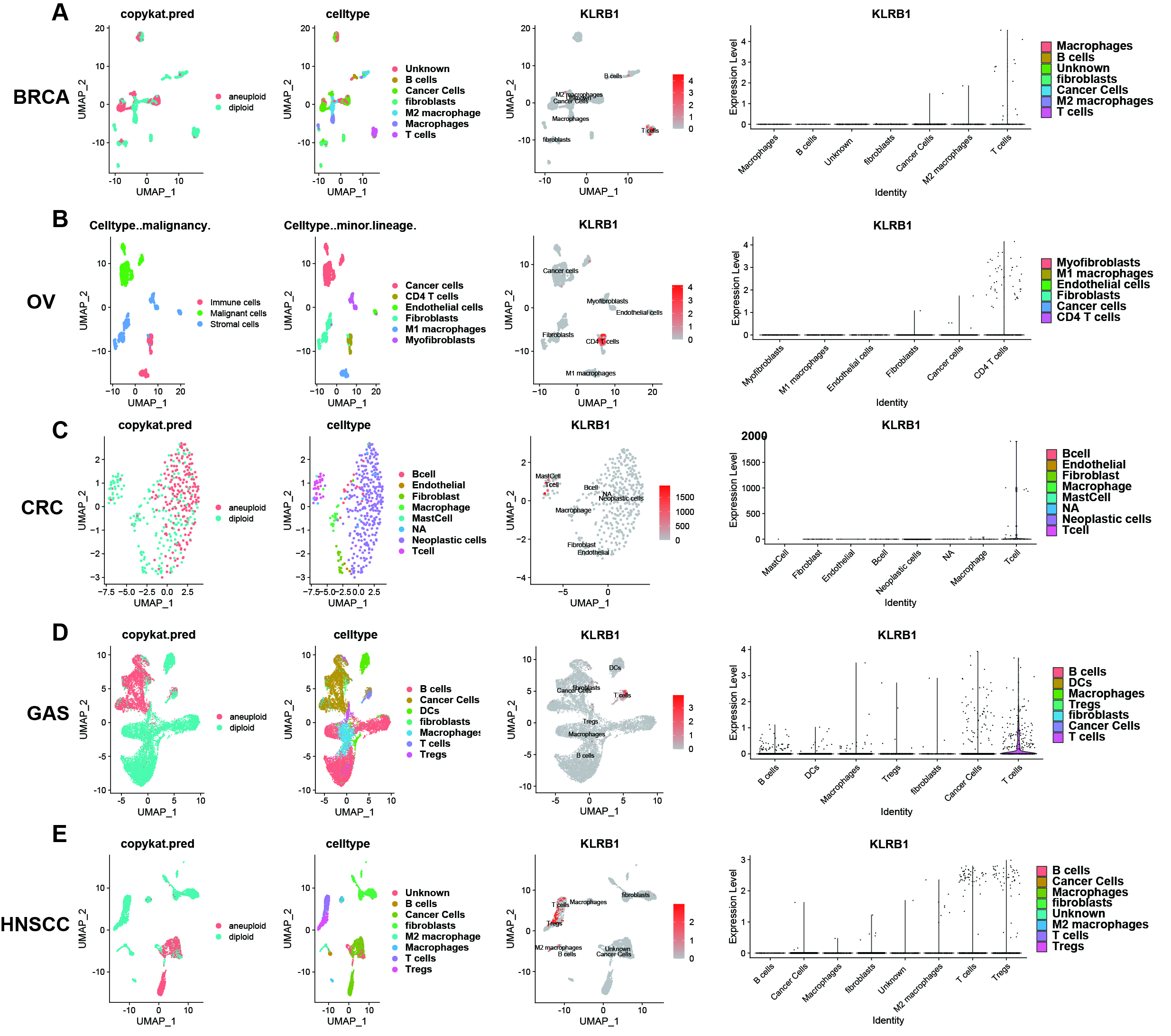


Figure S6. Single cell sequencing analysis the expression of CD161 on tumor and stromal cells. The expression levels of CD161 in tumor and stromal cells based on the R package copykat in BRCA (A), OV (B), CRC (C), GAS (D), HNSCC (E).


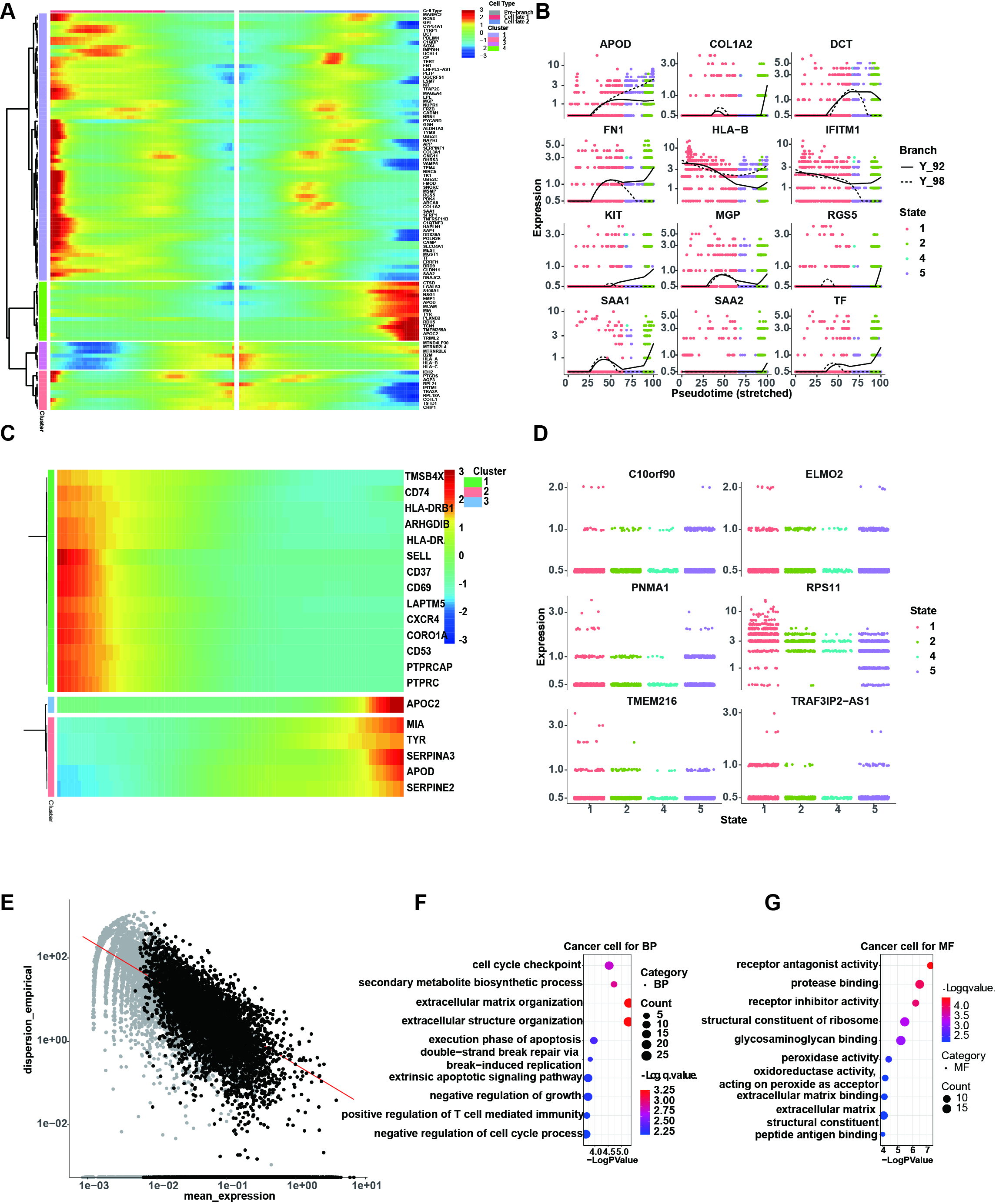


Figure S7. Single-cell pseudotime trajectories and functional annotations of cancer cells in SKCM. 100 genes with branch-dependent expression for branch point 1 were identified (A). the top 12 genes with branch-dependent expression for branch point 1 (B). Heatmap to display differentially expressed genes (DEGs) along the pseudotime trajectory (C). Pseudotime expression locus of differential genes (D). Tendency of dispersion between the expression of CD161 and cancer cells (E). Representative images of biological processes (BP) from GO enrichment analysis based on KLRB1 in cancer cells (F). Representative image of molecular functions (MF) from GO enrichment analysis based on KLRB1 in cancer cells (G).


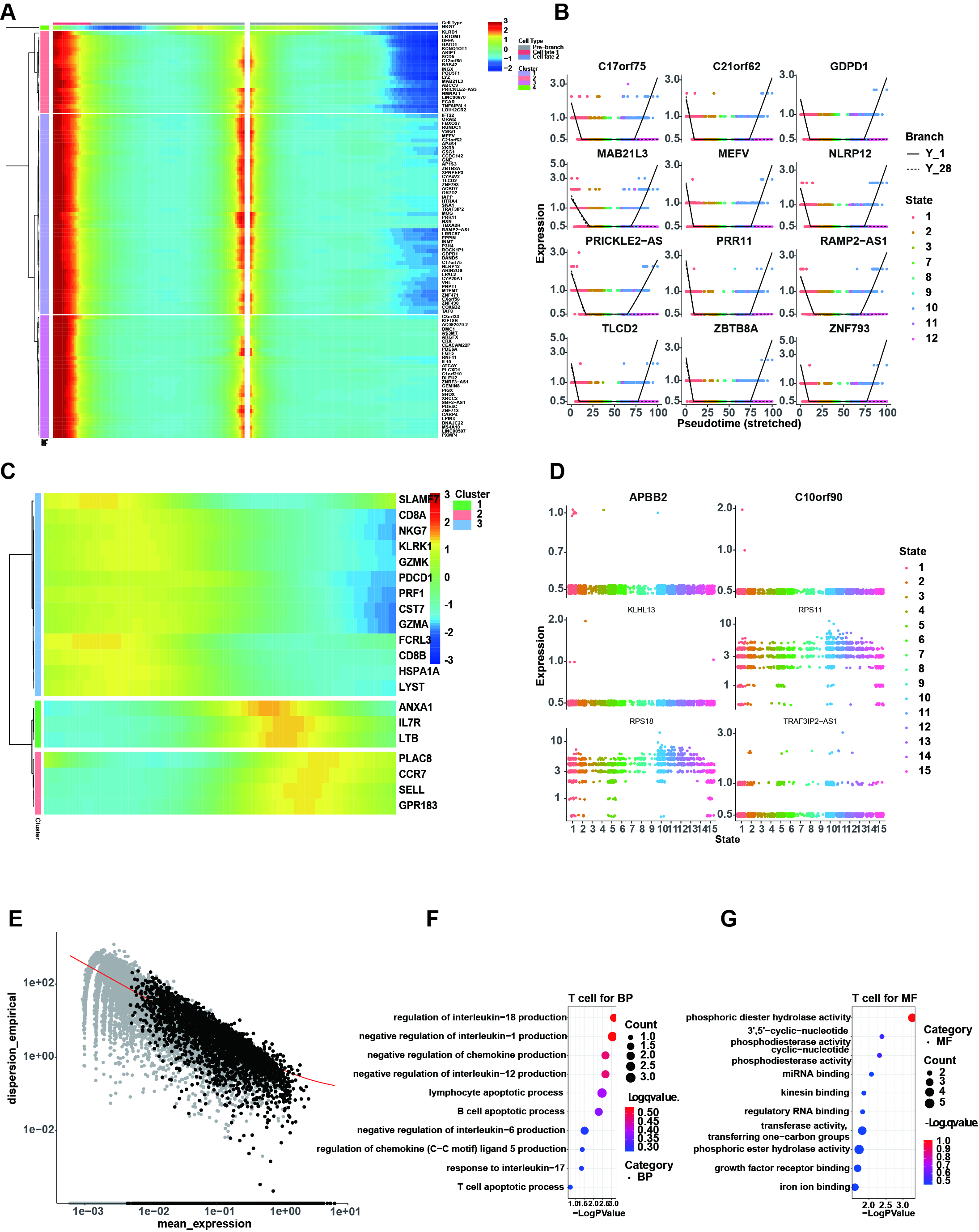


Figure S8. The Single-cell pseudotime trajectories and functional annotations of T cells in SKCM. 100 genes with branch-dependent expression for branch point 2 were identified (A). the top 12 genes with branch-dependent expression for branch point 2 (B). Heatmap to display differentially expressed genes (DEGs) along the pseudotime trajectory (C). Pseudotime expression locus of differential genes (D).Tendency of dispersion between the expression of CD161 and T cells (E). Representative image of biological processes (BP) from GO enrichment analysis based on KLRB1 in macrophage T cells (F). Representative image of molecular functions (MF) from GO enrichment analysis based on KLRB1 in T cells (G).


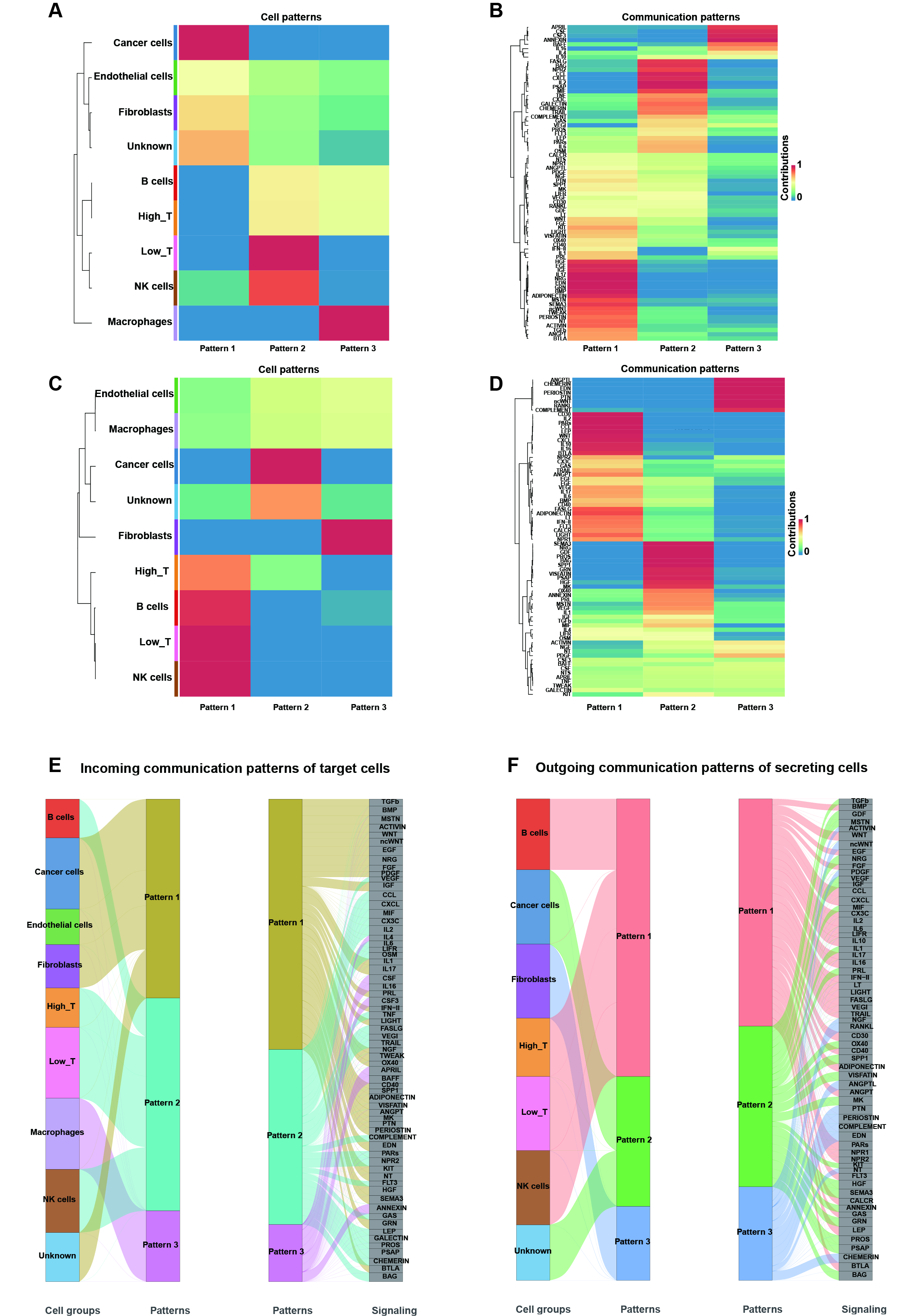


Figure S9. receiver of nine cell types(A) and related specific genes(B) showed three different cell patterns. senders of nine cell types(C) and related specific genes(D) showed three different cell patterns. The river plots depicted 9 cell types associated with the communication patterns of receiver(E) and sender(F).
